# Endoscopic balloon dilatation for benign hepaticojejunostomy anastomotic stricture using short double-balloon enteroscopy in patients with a prior Whipple’s procedure: a retrospective study

**DOI:** 10.1186/s12876-018-0742-x

**Published:** 2018-01-18

**Authors:** Sho Mizukawa, Koichiro Tsutsumi, Hironari Kato, Shinichiro Muro, Yutaka Akimoto, Daisuke Uchida, Kazuyuki Matsumoto, Takeshi Tomoda, Shigeru Horiguchi, Hiroyuki Okada

**Affiliations:** 1 Department of Gastroenterology, Okayama University Hospital, 2-5-1 Shikata-cho, Kita-ku, Okayama-city, Okayama 700-8558 Japan; 2 Department of Gastroenterology and Hepatology, Okayama University Graduate School of Medicine, Dentistry and Pharmaceutical Sciences, Okayama, Japan

**Keywords:** Choledochojejunostomy, Balloon dilatation, Double-balloon enteroscopy, Pancreatoduodenectomy

## Abstract

**Background:**

Endoscopic retrograde cholangiography using a short double-balloon endoscope (DB-ERC) is a promising minimally-invasive method for accessing hepaticojejunostomy (HJ) anastomosis in patients with surgically altered anatomy. We aimed to evaluate the immediate and long-term outcomes of balloon dilatation for benign HJ anastomotic stricture (HJAS) in patients who had previously undergone Whipple’s procedure using a DB-ERC.

**Methods:**

We conducted a retrospective analysis of 46 patients who underwent balloon dilatation alone with a DB-ERC for benign HJAS between November 2008 and November 2014. The median follow-up duration was 3.5 (interquartile range [IQR], 1.9–5.1) years.

**Results:**

The technical and clinical success rates were 100%, and adverse events occurred in 7% (3/46, cholangitis). The median hospitalization period was seven (IQR, 5–10) days. Of 42 patients (91%) followed-up for > 1 year, 24 (51%) had recurrent HJAS at a median of 1.2 (IQR, 0.6–2.9) years after balloon dilatation. The cumulative anastomotic patency rates at 1, 2, and 3 years were 73, 55, and 49%, respectively. In univariate analysis, early stricture formation (< 1 year) was a risk factor for recurrent stenosis, although no statistically significant risk factors were observed in multivariate analysis.

**Conclusions:**

Endoscopic balloon dilatation with DB-ERC for benign HJAS is effective and safe, having good immediate technical success and few adverse events. Further improvements to this procedure are needed to prevent recurrent HJAS.

## Background

Hepaticojejunostomy anastomotic stricture (HJAS) is an adverse event after pancreatoduodenectomy (PD) or pylorus-preserving pancreatoduodenectomy (PPPD) [1, 2] with Whipple’s procedure. HJAS is uncommon, occurring in 3–7% of patients 2.3–4.1 years after PD or PPPD [1, 2], but it is becoming more common because recent improvements in operative mortality rates for patients with pancreatobiliary cancer have led to an expansion of the operative indications to include various benign diseases such as chronic pancreatitis and intraductal papillary mucinous neoplasm (IPMN) [3]. Furthermore, chemotherapy has improved postoperative cancer survival. HJAS causes recurrent cholangitis, obstructive jaundice, stone formation, or liver abscess; treating HJAS and maintaining biliary flow is essential.

HJAS is treated using percutaneous or surgical procedures due to difficulties related to endoscopic access to the bile duct following gastrointestinal reconstruction. Percutaneous transhepatic procedures are associated with high success rates but several adverse events: sepsis, hemorrhage, pneumothorax, parietal pain, and restricted patient activity due to external drainage [4]. Surgical treatment for HJAS is more invasive and is often technically impossible if recurrent HJAS occurs.

Balloon enteroscopy facilitates diagnostic and therapeutic endoscopic retrograde cholangiopancreatography (ERCP) to treat postoperative pancreatobiliary disorders following gastrointestinal reconstruction [5–15]. Endoscopic methods such as balloon dilatation or plastic stent placement can now be performed for HJAS. However, no studies have reported the long-term effectiveness and safety of endoscopic balloon dilatation for HJAS.

We aimed to investigate the immediate and long-term outcomes of endoscopic balloon dilatation for HJAS following PD or PPPD using a short double-balloon enteroscope (DBE).

## Methods

### Patients

**Fig. 1:**
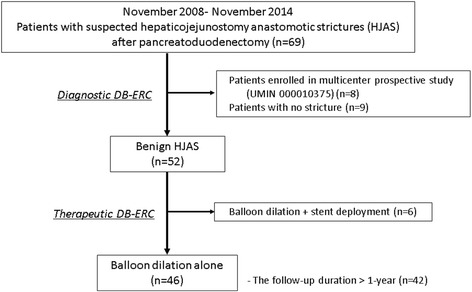
Flowchart of the patient enrollment process

Between November 2008 and November 2014, 69 patients with suspected benign HJAS after Whipple’s procedure underwent diagnostic endoscopic retrograde cholangiography using a short DBE (DB-ERC) at Okayama University Hospital (Fig. 1).

Whipple’s procedure involves classical PD, PPPD (which is PD with preservation of the pylorus in order to decrease postoperative dumping, marginal ulceration, and bile reflux gastritis), or subtotal stomach-preserving pancreatoduodenectomy (SSPPD) in which the pylorus ring is resected and most of the stomach is preserved in order to decrease delayed gastric emptying. PD, PPPD, or SSPPD was used in 30 (65%), 12 (26%), and 4 (9%) patients, respectively, and 45 patients (96%) received Braun anastomoses (Table 1).

**Table 1:** Patient characteristics

No. of patients	46
Median age, yrs. (IQR)	69 (64–75)
Gender, no (%)	
Male/Female	27 (59)/19(41)
Primary disease, no (%)	
Malignant disease	25 (54)
Pancreatic ductal cancer	12
Ampulla of vater cancer	4
Bile duct cancer	4
IPMC of the pancreas	2
Duodenal cancer	1
Renal cancer with pancreatic metastasis	1
Colon cancer with lymph node metastasis	1
Benign disease, no (%)	21 (46)
Pancreatic cystic tumor	14
IPMA/MCN/SPN	13/1/0
Chronic pancreatitis with obstructive jaundice	3
Pancreatic NET	2
Duodenal GIST	1
Ampulla of vater adenoma	1
Operative techique and reconstruction method, no (%)	
PD with/without Braun anastomosis	29/1 (63/2)
PPPD with Braun anastomosis	12 (26)
SSPPD with Braun anastomosis	4 (9)
Median time to occurrence of hepaticojejunostomy anastomotic stricture, year (IQR)	1.1 (0.6–2.9)

*IQR* interquartile range, *IPMC* intraductal papillary mucinous carcinoma, *IPMA* intraductal papillary mucinous adenoma, *MCN* mucinous cystic neoplasm, *SPN* solid pseudopapillary neoplasm, *NET* neruoendocrine tumor, *GIST* gastrointestinal stromal tumor, *PD* pancreatoduodenectomy, *PPPD* pylorus-preserving pancreatoduodenectomy, *SSPPD* subtotal stomach-preserving pancreatoduodenectomy

All patients had symptoms of cholestasis, cholangitis, or elevated hepatobiliary enzyme levels, and imaging findings of intrahepatic bile duct dilatation via abdominal ultrasonography, computed tomography (CT), or magnetic resonance cholangiopancreatography fulfilling The Tokyo Guidelines (TG13) [16].

Of 61 patients, excluding eight participating in a prospective multicenter study (UMIN 000010375) [6], 52 (85%) had benign HJAS. We defined benign HJAS as a pinpoint orifice or cicatricial mucosa without irregular papillogranular surface, nodular tumor, or irregular filling defect by cholangiography. The HJAS was also defined as an anastomosis diameter smaller than that of the distal bile duct, stagnant contrast medium flow after cholangiography, and cholangitis or cholestasis fulfilling TG13. Forty-six patients (75%) underwent endoscopic balloon dilatation alone for HJAS.

The information of this study was published on the internet. And Patients were given the opportunity to opt-out. This study was approved by the ethical committee of our hospital.

### Endoscopic procedures

DB-ERC was performed with a short DBE (EC-450BI5 or EI-530B, Fujifilm, Tokyo, Japan) with a 152-cm working length and a 2.8-mm working channel with carbon dioxide insufflation. Both devices had the same specifications. All procedures were performed by three experienced endoscopists who had performed more than 300 conventional ERCPs. Written informed consent was obtained from all patients.

**Fig. 2:**
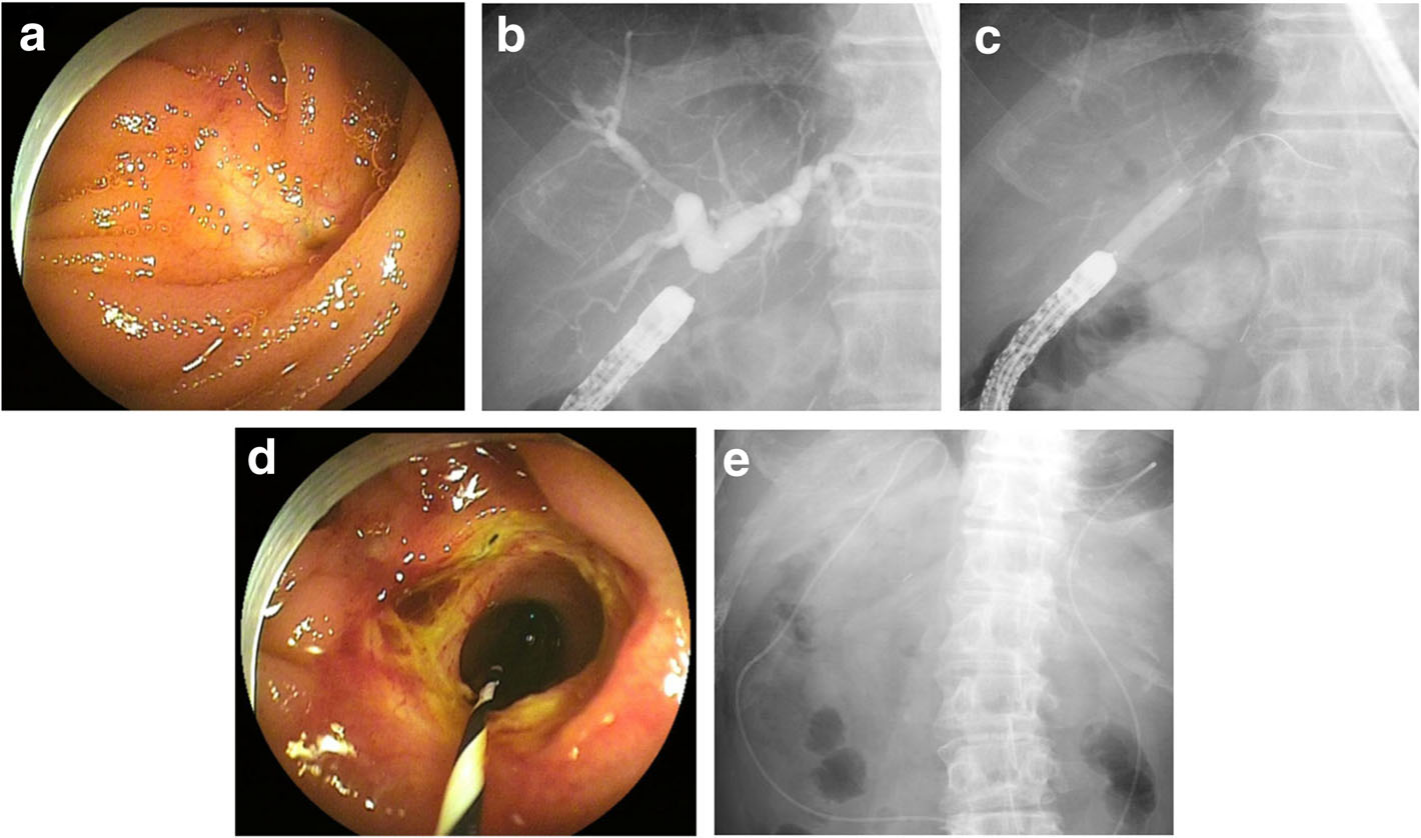
Endoscopic balloon dilatation for hepaticojejunostomy anastomotic stricture (HJAS) using a short double-balloon enteroscope. **a** The HJAS under direct vision. **b** Cholangiogram showing the dilated bile duct above the HJAS. **c** Fluoroscopic image of endoscopic balloon dilatation of the HJAS. **d** The open HJAS after endoscopic balloon dilatation. **e** Finally, a nasobiliary drainage tube was placed to avoid obstructive cholangitis

Patients were placed in the prone position and underwent conscious sedation with intravenous diazepam and pethidine. Butylscopolamine or glucagon was used for their antispasmodic effects. Two endoscopic approaches (the afferent-limb route and efferent-limb route) are possible for HJ anastomosis in patients with Braun anastomosis, and we usually selected the efferent-limb route because it allows accurate identification of the route to the HJ anastomosis. That is, selection of the middle of a three-pronged lumen at the Braun anastomosis for the route leads the scope to the HJ anastomosis and allows for better maneuverability [7]. After reaching close to the blind end of the jejunum, endoscopic visualization of the pinpoint orifice or cicatricial mucosa was attempted to identify the HJ anastomosis, sometimes using fluoroscopic guidance (Fig. 2a). Biliary cannulation and cholangiography were generally performed using a 4-Fr tapered catheter (PR-V234Q, Olympus, Tokyo, Japan) and a 0.025–0.035-in. guidewire (Visiglide, Olympus; Revowave, Piolax, Yokohama, Japan) (Fig. 2b). After inserting the guidewire, a 6–8-mm diameter balloon dilatation catheter (Maxpass, Olympus; Quantum TTC, COOK, Tokyo, Japan) matching the diameter of the bile duct above the HJAS was inserted through the stricture. The balloon was inflated at the stricture for 30–60 s to achieve complete dilatation (maximum pressure = 8 atm) (Fig. 2c, d) [13, 15, 17]. In some cases involving intrahepatic bile duct stones, an endoscopic biliary stone extraction was performed using a retrieval balloon catheter, basket catheter, and mechanical lithotripter.

If a biliary cannulation or guidewire insertion was difficult because of severe stricture, a 3-Fr tapered catheter (Contour 5–4-3 tip, Boston Scientific, Tokyo, Japan) and a thinner 0.018-in. guidewire (Roadrunner, COOK) were used. In addition, if advancement of dilatation catheters through a severe stricture was difficult despite successful guidewire insertion, a 7-Fr screw-type device (Soehendra Stent Retriever, COOK) or a 7-Fr Soehendra biliary dilatation catheter (COOK) was used for pre-dilatation of the anastomosis over the guidewire [8].

Following balloon dilatation, an endoscopic nasobiliary drainage (ENBD) tube was placed to treat concomitant cholangitis and to avoid obstructive cholangitis due to edema of the HJ anastomosis (Fig. 2e). A few days later, the outflow of contrast medium through the anastomosis was confirmed through cholangiography using an ENBD catheter, and the catheter was removed.

### Follow-up

Antibiotics were administered for at least 2 days, including the day of treatment initiation. Dietary intake was started 1 day after the procedure when a good clinical course was confirmed. Patients were followed at 3–6-month intervals on an outpatient basis with laboratory tests. Furthermore, imaging was performed when elevated hepatobiliary enzyme levels were revealed or patients reported symptoms suggestive of cholangitis. If necessary, objective information was also collected by contacting the patient’s family doctor.

### Study definitions

Diagnostic technical success was defined as successful scope insertion to the HJ anastomosis, identification of the anastomosis, biliary cannulation, and cholangiography. Therapeutic technical success was defined as successful balloon dilatation for HJAS with or without the removal of intrahepatic bile duct stones. Scope insertion time was defined as the time from scope insertion to when the HJ anastomosis was reached. Procedural time was defined as the time from scope insertion until withdrawal. Clinical success was defined as sustained improvement in symptoms and laboratory parameters after balloon dilatation. Adverse events were defined according to the ASGE guidelines [18]. In addition, recurrent HJAS was defined as both clinically documented and DB-ERC-documented recurrence of HJAS after initial balloon dilatation. Duration of HJ anastomotic patency was defined as the time from the date of balloon dilatation to the date of HJAS recurrence. The follow-up duration was defined as the time from the date of balloon dilatation to the date of the last visit.

### Statistical analysis

Continuous data are presented as medians and interquartile ranges. Cumulative anastomotic patency after balloon dilatation was assessed using the Kaplan-Meier method. Continuous variables were categorized into two groups by the median. To identify risk factors for recurrent HJAS > 1 year after balloon dilatation, factors with *P*-values < 0.20 in a univariate Cox proportional hazard model were analyzed in a multivariate Cox model. The following variables were analyzed: sex, age, preoperative factors (body mass index, common bile duct diameter, and C-reactive protein level), pre-endoscopic treatment factors (intrahepatic bile duct stones, cholangitis, serum albumin level, and earlier HJAS after operation [< 1 year], anastomotic leakage, and pancreatic fistulae after the previous PD), and factors during endoscopic treatment (remaining balloon waste during inflation and endoscopic biliary stone extraction). Hazard ratios and 95% confidence intervals (CIs) were calculated for each factor. A P-value < 0.05 was considered statistically significant. All statistical analyses were performed with JMP software (ver.11; SAS Institute, Cary, NC, USA) except for the Kaplan-Meier method, which was performed using GraphPad Prism 6.0 (Graph Pad Software, San Diego, CA).

## Results

### Patient characteristics

Patient characteristics are summarized in Table 1. Of 46 patients with HJAS, 25 (54%) underwent Whipple’s procedure for malignant diseases (mainly pancreatic ductal cancer). The remaining 21 patients (46%) underwent surgery for benign diseases (mainly IPMN). All patients experienced HJAS for the first time; the median time-to-HJAS was 1.1 (0.6–2.9) years.

### Immediate outcomes: technical success, clinical success, and adverse events

The immediate outcomes are shown in Table 2. All 46 patients achieved diagnostic and therapeutic technical success. A thinner catheter was required for biliary cannulation in two patients (4%) due to severe HJAS. For pre-dilatation before biliary cannulation and balloon dilatation, a 7-Fr screw-type device and a 7-Fr SBDC were required in one patient (2%) and seven patients (15%), respectively. In addition, endoscopic biliary stone extraction was performed in 12 patients (26%). An ENBD tube was placed correctly in 38 patients (83%). Cholangiography, using an ENBD tube, revealed residual stones in four patients. These were removed by additional treatment. The median period of ENBD tube placement was two (2–4) days; accidental removal occurred in one patient.

**Table 2:** Details of endoscopic treatment

The diagnostic and therapeutic technical success, no (%)	46/46 (100)
Median time of scope insertion, min (IQR)	13 (7–29)
Median procedural time, min (IQR)	54 (37–82)
Biliary cannulation, no (%)	
Standard catheter	44 (96)
Thinner catheter	2 (4)
Pre-dilation, no (%)	
7-Fr screw-type device for biliary cannulation	1 (2)
7-Fr SBDC for balloon dilation	7 (15)
Balloon dilator, no (%)	
6-mm in diameter	7 (15)
8-mm in diameter	39 (85)
Endoscopic biliary stone extraction, no (%)	12 (26)
Endoscopic nasobiliary drainage, no (%)	38 (83)
Hospitalization, day (IQR)	7 (5–10)

*IQR* interquartile range, *SBDC* Soehendra biliary dilation catheter

Thereafter, all patients achieved clinical success. The median time of scope insertion was 13 (7–29) min, and the median procedural time was 54 (37–82) min. The median hospitalization time was seven (5–10) days.

The adverse events rate was 7% (3/46) for cholangitis. There were no serious adverse events, including perforation or pancreatitis. All three patients improved with conservative medical treatment.

### Long-term outcomes: hepaticojejunostomy anastomotic patency after endoscopic balloon dilatation

**Fig. 3:**
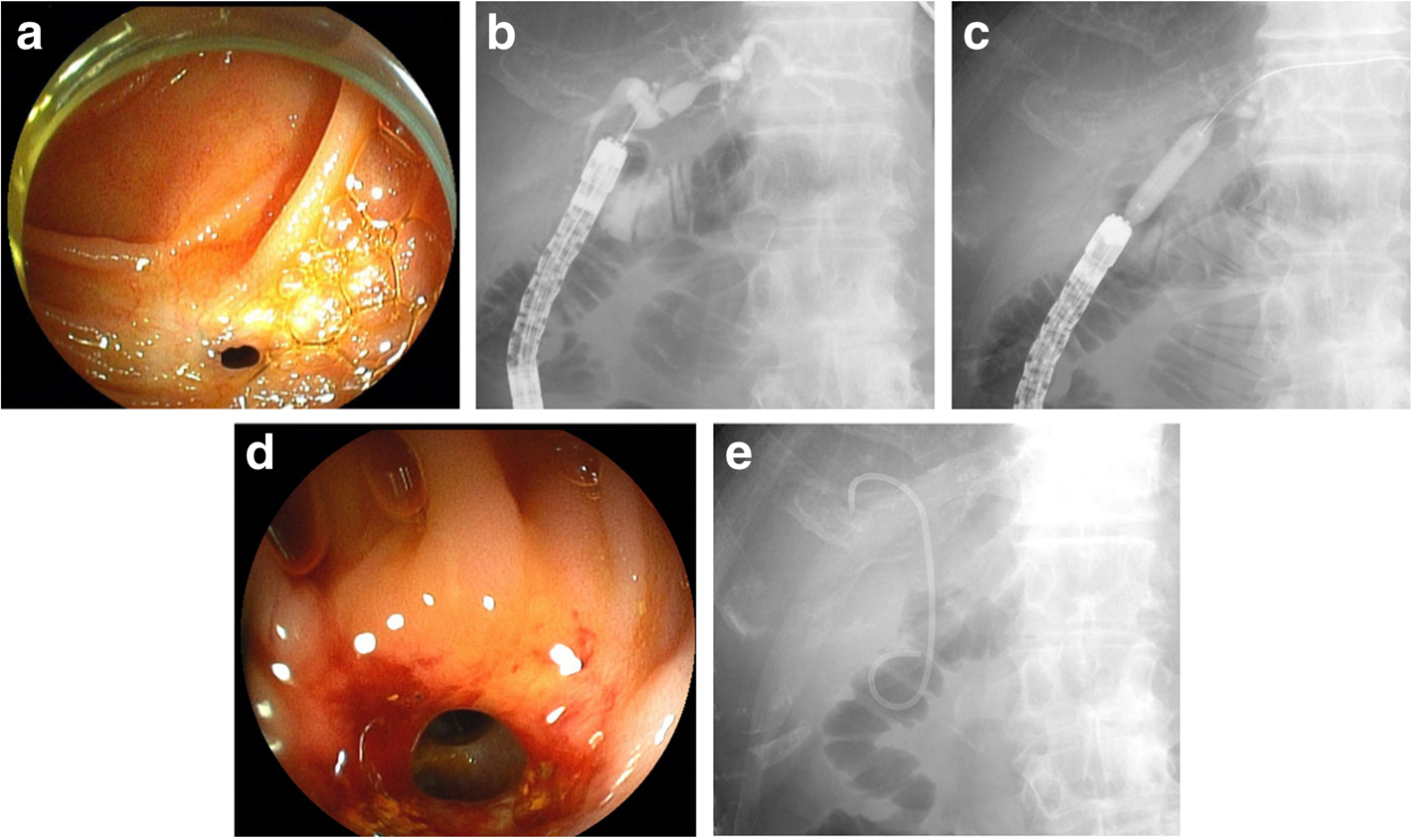
Endoscopic balloon dilatation for recurrent HJAS (same case as shown in Fig. 2). **a** The recurrent HJAS occurred 5 years after endoscopic balloon dilatation. **b** Cholangiogram showing the dilated bile duct above the recurrent HJAS. **c** Fluoroscopic image of endoscopic balloon dilatation of the recurrent HJAS. **d** The open recurrent HJAS after endoscopic balloon dilatation. **e** A plastic stent placed across the HJAS. Fluoroscopic image after stent placement

The median follow-up duration after balloon dilatation was 3.5 (1.9–5.0) years. In 42 patients (91%) with a follow-up period > 1 year, 24 (57%) developed recurrent HJAS at a median of 1.2 (0.6–2.9) years after balloon dilatation. All patients with recurrent HJAS underwent endoscopic treatment with DB-ERC (Fig. 3) and achieved technical and clinical success (Table 3). Balloon dilatation alone, stent deployment alone, and balloon dilatation with stent deployment were performed in 9 patients (38%), 1 patient (4%), and 14 patients (58%), respectively. An endoscopic biliary stone extraction was performed in 11 patients (46%). Thereafter, stent removal was performed in 80% (12/15), and the median duration of stent placement was 232 (147–324) days.

**Table 3:** Details of endoscopic treatment of recurrent HJAS

The therapeutic technical success, no (%)	24/24 (100)
Median procedural time, min (IQR)	46 (39–62)
Treatment method, no (%)	
Balloon dilatation only	9 (38)
stent deployment only	1 (4)
Balloon dilatation + stent deployment	14 (58)
Endoscopic biliary stone extraction, no (%)	11 (46)
Hospitalization, day (IQR)	5 (4–8)
Stent free patients, no (%)	12/15 (80)
Duration of stent placement, day (IQR)	232 (147–324)

*IQR* interquartile range

**Fig. 4:**
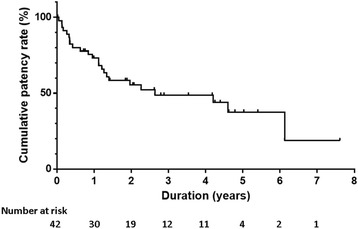
Cumulative patency rates of hepaticojejunostomy anastomotic stricture after endoscopic balloon dilatation by the Kaplan-Meier method

The cumulative anastomotic patency rates at 1, 2, and 3 years were 73% (95% CI, 58–84), 55% (95% CI, 39–69), and 49% (95% CI, 32–63), respectively (Fig. 4).

### Risk factors for recurrence of hepaticojejunostomy anastomotic stricture after endoscopic balloon dilatation

In univariate analysis, early stricture formation (< 1 year) was a risk factor for recurrent HJAS, although not in multivariate analysis.

Therefore, we found no statistically significant risk factors in multivariate analysis for recurrent HJAS after balloon dilatation (Table 4).

**Table 4:** The Risk Factors of recurrent hepaticojejunostomy anastomotic stricture after endoscopic balloon dilatation

				Recurence		Univariate analysis			Multivariate analysis		
			All	(+)	(−)	HR	95% CI	*P* value	HR	95% CI	*P* value
Patient’s factors	Sex	male	25	11	14	2.08	(0.92–4.75)	0.076	2.00	(0.86–4.77)	0.108
		female	17	13	4						
	Age (years)	≧65	30	18	12	0.65	(0.24–1.57)	0.356			
		< 65	12	6	6						
Preoperative factors	BMI	≧25	23	13	10	1.39	(0.61–3.13)	0.427			
		< 25	19	11	8						
	Diameter of common bile duct (mm)	≧7	27	12	15	1.81	(0.80–4.11)	0.150	1.62	(0.67–3.90)	0.276
		< 7	15	12	3						
	CRP (mg/dl)	≧1.00	15	9	6	0.96	(0.42–2.29)	0.919			
		< 1.00	27	15	12						
Pre-endoscopic treatment factors	Intrahepatic bile duct stones	(+)	9	3	6	1.91	(0.65–8.12)	0.260			
		(−)	33	21	12						
	Cholangitis	(+)	24	14	10	1.23	(0.52–2.81)	0.623			
		(−)	18	10	8						
	Serum albumin level (normal ≧3.5 mg/dl)	≧3.5	13	6	7	1.06	(0.44–2.96)	0.896			
		< 3.5	29	18	11						
	Earlier stricture after operation (< 1 year)	(+)	20	15	5	2.32	(1.03–5.56)	0.043	1.72	(0.72–4.31)	0.226
		(−)	22	9	13						
	Biliary anastomotic leakage after operation	(+)	1	1	0	9.74	(0.50–65.9)	0.107	13.2	(0.62–107)	0.084
		(−)	41	23	18						
	Pancreatic fistulae after operation	(+)	20	10	10	0.74	(0.32–1.66)	0.469			
		(−)	22	14	8						
Factors during endoscopic treatment	Remaining balloon waste during inflation	(+)	4	3	1	0.49	(0.17–2.11)	0.301			
		(−)	38	21	17						
	Endoscopic biliary stone extraction	(+)	11	4	7	1.82	(0.68–6.30)	0.246			
		(−)	31	20	11						

*HR* hazard ratio, *CI* confidence interval, *BMI* body mass index, *CRP* C-reactive protein

## Discussion

HJAS treatment aims to remove the stricture and maintain biliary flow. Access to the HJAS is achieved using endoscopic, percutaneous, or surgical procedures. Advances in percutaneous and endoscopic interventional techniques have caused a shift from open to minimally-invasive methods [19]. The success rate of the percutaneous approach is high (91–100%) in patients with surgically altered anatomy [1, 2, 20–26]. The advantage of percutaneous transhepatic procedures is that once the route is established, the bile duct is accessed easily. This is useful when repeated procedures are required. However, initial drainage is usually achieved via an external drain, which negatively affects the patient’s activity and quality of life. Furthermore, the anatomical characteristics of the HJ site sometimes cause difficulties in advancing the guidewire through the HJAS and securing biliary flow to the opposite side of the puncture. Therefore, percutaneous transhepatic cholangioscopic guidance for targeted recanalization with a guidewire [27] and two punctures for complete drainage are often needed.

In endoscopic procedures, if only the scope reaches the HJ anastomosis, biliary cannulation can be attempted under direct visualization. In previous studies, the rate of successful ERCP for biliary indications following PD or PPPD was 69–86% using conventional endoscopes, such as a front-viewing enteroscope, pediatric colonoscope, or duodenoscope [28–30]. However, even in patients requiring repeated ERCPs, the same instrument may not be successful each time due to excessive looping of the endoscope, excessive afferent loop length, or afferent loop adhesions. The short DBE increased this rate to 97% (31 of 32 sessions) in 20 prior-PD patients [9–12] and 95% in another study (71 of 75 prior-PD or PPPD patients) [6]. A high success rate (100%, 46 of 46 patients) was achieved in the present study. Therefore, the short DBE is a useful biliary access tool for patients who have undergone PD or PPPD and can be considered as the instrument of first-choice by experienced endoscopists. Percutaneous transhepatic procedures should be reserved for cases of failed or unfeasible ERCP.

The most commonly used treatment for HJAS is balloon dilatation, which can be performed percutaneously or endoscopically. Percutaneous balloon dilatation with temporary internal or external stent placement is effective. Bonell and colleagues reported that the one, four, and six-year patency rates were 97, 96, and 96%, respectively using a 10-mm balloon and a 14-Fr stent for a median of 8 months [24]. Cantwell and colleagues reported a five-year patency rate of 53% using a 10–12-mm balloon and a 10–12-Fr stent for a mean of 1 month [25]. Based on previous reports [20–23, 26], percutaneous transhepatic dilatation for HJAS requires long-term indwelling stents for acceptable results, although there is no consensus regarding balloon size, stent size, number of dilatations, pressure level during dilatation, management of residual balloon waist (response evaluation to balloon dilatation), and duration of stenting after dilatation.

Endoscopic biliary intervention for HJAS using a short DBE is effective and safe [5–15], but the long-term outcomes are not clear. We assessed the usefulness of endoscopic balloon dilatation alone for treating HJAS. However, this endoscopic treatment did not provide favorable long-term outcomes (cumulative anastomotic patency rates at 1 and 3 years were 73% and 49%, respectively) despite high technical success and low adverse events rates. In our previous report on endoscopic treatment using a short DBE for HJAS after living-donor liver transplantation (LT), the period of bile duct patency was significantly longer in the balloon dilatation + biliary stent group (*n* = 7) than that in the balloon dilatation alone group (*n* = 7) during a median follow-up of 22 months (*P* = 0.017) [13]. In addition, the most common therapy for benign biliary strictures with duct-to-duct reconstruction after LT involves endoscopic balloon dilatation and placement of multiple plastic stents followed by periodic replacement of the stents for approximately 1 year to allow for expansion and remodeling of the strictures [17, 31–35]. Although these anastomotic strictures after LT, which result from ischemia, fibrotic healing, and surgical technique, may be different from HJAS after PD or PPPD, balloon dilatation combined with stent deployment might be recommended for definite resolution and maintenance of biliary flow in patients who have previously undergone PD and PPPD. Further study is needed to determine the usefulness of these combined therapies, especially considering their drawbacks, such as the need for repeated ERCPs for stent exchange and the risk of cholangitis resulting from stent occlusion.

Risk factors for recurrent HJAS after percutaneous or endoscopic treatment in patients who have previously undergone prior PD or PPPD have not been reported. We did not identify any statistically significant risk factors in multivariate analysis. Because it is impossible to predict which patients will develop recurrent HJAS and initial balloon dilatation alone can be effective for 3 years in approximately 50% of patients, balloon dilatation alone might be considered as first-line treatment for HJAS in patients who have previously undergone PD or PPPD.

In the present study, 7% of patients experienced adverse events (cholangitis). In all cases, the situation was resolved with conservative treatment. In a previous report, while adverse events such as hemorrhage, cholangitis, and hemorrhagic pleural effusion occurred in 10–24% patients treated by percutaneous transhepatic procedures [20–22, 24, 26], biliary damage and microperforation of the HJ anastomosis occurred in just 4% of patients (3 of 75 patients with prior PD or PPPD) treated by endoscopic procedures using a short DBE [6]. All three patients recovered with conservative treatment, but excessive balloon dilatation using a larger balloon caused biliary laceration. Therefore, endoscopic procedures are less invasive than percutaneous transhepatic procedures and choosing the optimal balloon size to match the bile duct above the HJAS is crucial.

This study had some limitations. First, it was retrospective and performed at a single center. Second, the number of patients was small. Third, anastomotic patency might have been overestimated because three (50%) of the six excluded patients underwent stent placement due to membranous obstruction with severe cholangitis. Fourth, recurrent HJAS might have been underestimated because asymptomatic outpatients are not suspected of having recurrent HJAS unless they attend the hospital and undergo laboratory tests or imaging studies. In future studies, patients should be followed-up at shorter intervals to identify and treat HJAS in patients at risk of serious hepatobiliary disorders.

## Conclusions

Endoscopic balloon dilatation using a short DBE provides favorable short-term results for the treatment of benign HJAS in patients who have previously undergone PD or PPPD if performed by experienced endoscopists. Despite these immediate results, unfavorable long-term outcomes were observed with a high rate of recurrent HJAS. A strategy for treating HJAS using a short DBE while providing better long-term outcomes is required.

## Abbreviations

ASGE: American Society for Gastrotinestinal Endoscopy
BMI: Body mass index
CIs: Confidence intervals
CRP: C-reactive protein
DBE: Double-balloon enteroscope
DB-ERC: Endoscopic retrograde cholangiography using a short double-balloon endoscope
ENBD: Endoscopic nasobiliary drainage
ERCP: Endoscopic retrograde cholangiopancreatography
GIST: Gastrointestinal stromal tumor
HJ: Hepaticojejunostomy
HJAS: HJ anastomotic stricture
HR: Hazard ratio
IPMA: Intraductal papillary mucinous adenoma
IPMC: Intraductal papillary mucinous carcinoma
IPMN: Intraductal papillary mucinous neoplasm
IQR: Interquartile range
LT: Liver transplantation
MCN: Mucinous cystic neoplasm
NET: Neruoendocrine tumor
PD: Pancreatoduodenectomy
PPPD: Pylorus-preserving pancreatoduodenectomy
SBDC: Soehendra biliary dilation catheter
SP: Solid pseudopapillary neoplasm
SSPPD: Stomach-preserving pancreatoduodenectomy
TG13: Tokyo Guidelines 2013

## References

[1] Reid-Lombardo KM, Ramos-De la Medina A, Thomsen K (2007). Long-term anastomotic complications after pancreaticoduodenectomy for benign diseases. J Gastrointest Surg.

[2] House MG, Cameron JL, Schulick RD (2006). Incidence and outcome of biliary strictures after pancreaticoduodenectomy. Ann Surg.

[3] Balcom JH, Rattner DW, Warshaw AL (2001). Ten-year experience with 733 pancreatic resections: changing indications, older patients, and decreasing length of hospitalization. Arch Surg.

[4] Winick AB, Waybill PN, Venbrux AC (2001). Complications of percutaneous transhepatic biliary interventions. Tech Vasc Interv Radiol.

[5] Park BK, Jeon TJ, Jayaraman V (2016). Endoscopic retrograde Cholangiopancreatography in patients with previous Pancreaticoduodenectomy: a single-center experience. Dig Dis Sci.

[6] Shimatani M, Hatanaka H, Kogure H (2016). Diagnostic and therapeutic endoscopic retrograde Cholangiography using a short-type double-balloon endoscope in patients with altered gastrointestinal anatomy: a multicenter prospective study in Japan. Am J Gastroenterol.

[7] Tsutsumi K, Kato H, Muro S (2015). ERCP using a short double-balloon enteroscope in patients with prior pancreatoduodenectomy: higher maneuverability supplied by the efferent-limb route. Surg Endosc.

[8] Tsutsumi K, Kato H, Sakakihara I (2013). Dilation of a severe bilioenteric or pancreatoenteric anastomotic stricture using a Soehendra stent retriever. World J Gastrointest Endosc.

[9] Shimatani M, Matsushita M, Takaoka M (2009). Effective “short” double-balloon enteroscope for diagnostic and therapeutic ERCP in patients with altered gastrointestinal anatomy: a large case series. Endoscopy.

[10] Osoegawa T, Motomura Y, Akahoshi K (2012). Improved techniques for double-balloon-enteroscopy-assisted endoscopic retrograde cholangiopancreatography. World J Gastroenterol.

[11] Cho S, Kamalaporn P, Kandel G (2011). ‘Short’ double-balloon enteroscope endoscopic retrograde cholangiopancreatography in patients with a surgically altered upper gastrointestinal tract. Can J Gastroenterol.

[12] Tsujino T, Yamada A, Isayama H (2010). Experiences of biliary interventions using short double-balloon enteroscopy in patients with roux-en-Y anastomosis or hepaticojejunostomy. Dig Endosc.

[13] Tomoda T, Tsutsumi K, Kato H (2016). Outcomes of management for biliary stricture after livingdonor liver transplantation with hepaticojejunostomy using short-type double-balloon enteroscopy. Surg Endosc.

[14] Sanada Y, Mizuta K, Yano T (2011). Double-balloon enteroscopy for bilioenteric anastomotic stricture after pediatric living donor liver transplantation. Transpl Int.

[15] Itokawa F, Itoi T, Ishii K (2014). Single- and double-balloon enteroscopy-assisted endoscopic retrograde cholangiopancreatography in patients with roux-en-Y plus hepaticojejunostomy anastomosis and Whipple resection. Dig Endosc.

[16] Takada T, Strasberg SM, Solomkin JS (2013). TG13: updated Tokyo guidelines for the management of acute cholangitis and cholecystitis. J Hepatobiliary Pancreat Sci.

[17] Zoepf T, Maldonado-Lopez EJ, Hilgard P (2006). Balloon dilatation vs. balloon dilatation plus bile duct endoprostheses for treatment of anastomotic biliary strictures after liver transplantation. Liver Transpl.

[18] Cotton PB, Eisen GM, Aabakken L (2010). A lexicon for endoscopic adverse events: report of an ASGE workshop. Gastrointest Endosc.

[19] Millis JM, Tompkins RK, Zinner MJ (1992). Management of bile duct strictures. An evolving strategy. Arch Surg.

[20] Kim JH, Lee SK, Kim MH (2003). Percutaneous transhepatic cholangioscopic treatment of patients with benign bilio-enteric anastomotic strictures. Gastrointest Endosc.

[21] Schumacher B, Othman T, Jansen M (2001). Long-term follow-up of percutaneous transhepatic therapy (PTT) in patients with definite benign anastomotic strictures after hepaticojejunostomy. Endoscopy.

[22] Weber A, Rosca B, Neu B (2009). Long-term follow-up of percutaneous transhepatic biliary drainage (PTBD) in patients with benign bilioenterostomy stricture. Endoscopy.

[23] Glas L, Courbière M, Ficarelli S (2008). Long-term outcome of percutaneous transhepatic therapy for benign bilioenteric anastomotic strictures. J Vasc Interv Radiol.

[24] Bonnel DH, Fingerhut AL (2012). Percutaneous transhepatic balloon dilatation of benign bilioenteric strictures: long-term results in 110 patients. Am J Surg.

[25] Cantwell CP, Pena CS, Gervais DA (2008). Thirty years’ experience with balloon dilation of benign postoperative biliary strictures: long-term outcomes. Radiology.

[26] Köcher M, Cerná M, Havlík R (2007). Percutaneous treatment of benign bile duct strictures. Eur J Radiol.

[27] Yang DH, Lee SK, Moon SH (2009). Percutaneous transhepatic cholangioscopic intervention in the management of complete membranous occlusion of bilioenteric anastomosis: report of two cases. Gut Liver.

[28] Chahal P, Baron TH, Topazian MD (2006). Endoscopic retrograde cholangiopancreatography in post-Whipple patients. Endoscopy.

[29] Farrell J, Carr-Locke D, Garrido T (2006). Endoscopic retrograde cholangiopancreatography after pancreaticoduodenectomy for benign and malignant disease: indications and technical outcomes. Endoscopy.

[30] Matsushita M, Takakuwa H, Uchida K (2009). Techniques to facilitate ERCP with a conventional endoscope in patients with previous pancreatoduodenectomy. Endoscopy.

[31] Tabibian JH, Asham EH, Han S (2010). Endoscopic treatment of postorthotopic liver transplantation anastomotic biliary strictures with maximal stent therapy (with video). Gastrointest Endosc.

[32] Hsieh TH, Mekeel KL, Crowell MD (2013). Endoscopic treatment of anastomotic biliary strictures after living donor liver transplantation: outcomes after maximal stent therapy. Gastrointest Endosc.

[33] Rerknimitr R, Sherman S, Fogel EL (2002). Biliary tract complications after orthotopic liver transplantation with choledochocholedochostomy anastomosis: endoscopic findings and results of therapy. Gastrointest Endosc.

[34] Kao D, Zepeda-Gomez S, Tandon P (2013). Managing the post-liver transplantation anastomotic biliary stricture: multiple plastic versus metal stents: a systematic review. Gastrointest Endosc.

[35] Tringali A, Barbaro F, Pizzicannella M (2016). Endoscopic management with multiple plastic stents of anastomotic biliary stricture following liver transplantation: long-term results. Endoscopy.

